# Can Contrast Injections Cause or Propagate Coronary Injuries? Insights From Vessel and Guiding Catheter Hemodynamics

**DOI:** 10.1016/j.jscai.2024.102396

**Published:** 2024-10-29

**Authors:** Daniel Chamié, Rahul Bahl, Julio Maia, Mauro Echavarria-Pinto, Suraya Gafore, Amr Saleh, Ecaterina Cristea, Henry Seligman, Rodrigo M. Joaquim, Fausto Feres, Sayan Sen, Rasha Al-Lamee, Marinella Centemero, Christopher Baker, Tom Johnson, Matthew J. Shun-Shin, Alexandra J. Lansky, Ricardo Petraco

**Affiliations:** a Section of Cardiovascular Medicine, Yale School of Medicine, New Haven, Connecticut; b Instituto Dante Pazzanese de Cardiologia, São Paulo, Brazil; c Imperial College London, United Kingdom; d CEDIPAR, Maringá, Brazil; e Hospital General ISSSTE Querétaro, Querétaro, Mexico; f Bristol Heart Institute, Translational Health Sciences, University of Bristol, Bristol, United Kingdom

**Keywords:** chronic total occlusions, contrast, coronary angiography, coronary dissections, optical coherence tomography

## Abstract

**Background:**

The mechanistic association between the hydraulic forces generated during contrast injection and the risk of coronary injury is poorly understood. In this study, we sought to evaluate whether contrast injections increase intracoronary pressures beyond resting levels and estimate the risk of hydraulic propagation of coronary dissections.

**Methods:**

This is a prospective, single-arm, multicenter study that included patients with nonculprit, non−flow-limiting coronaries. A continuous 60-second pressure recording was taken at 5 predetermined locations during contrast injections: distal, mid, and proximal vessel, catheter tip, and inside the catheter. The primary end point was the change in intracoronary peak pressure between resting and injections in each location.

**Results:**

A total of 269 pressure recordings (58 vessels; 52 patients) were analyzed. Injections led to a small increase in peak pressure in the distal (mean difference, +4.5 mm Hg; 95% CI, 1.5-7.4), mid (mean difference, +4.1 mm Hg; 95% CI, 1.4-6.9), and proximal (mean difference, +5.1 mm Hg; 95% CI, 2.5-7.7) vessel locations, and much higher increases at the catheter tip (mean difference, +11.7 mm Hg; 95% CI, 5.8-17.7) and inside the catheter (mean difference, +77.5 mm Hg; 95% CI, 64.5-90.4). Compared to the distal vessel, pressure changes were only significant at the catheter tip (+10 mm Hg; *P* < .01) and inside the catheter (+79.1 mm Hg; *P* < .01).

**Conclusions:**

Contrast injections lead to negligible changes in intracoronary pressures beyond the catheter tip. Although it is sensible to minimize injections when coronary dissections are close to the catheter, it is unlikely that they would cause injuries beyond the catheter tip.

## Introduction

Coronary artery dissection is a recognized mechanism of arterial injury that can occur spontaneously, during intracoronary instrumentation, or as a consequence of trauma to the chest.^1^, ^2^, ^3^, ^4^ There is a widely perceived idea that the hydraulic forces generated by contrast injections during angiography (pressure applied by hand or automated injectors) can either cause or propagate coronary dissections.^5^ This mechanistic association between injection and injury is intuitively supported by the recognized risk of ventricular arrhythmias when coronary injections are performed into a small coronary branch (commonly the right-sided conus artery).^6^ Therefore, when a coronary dissection is suspected or confirmed, consensus-based practice recommends physicians limit the use of contrast injections to avoid worsening its severity and potentially putting the patient at risk of arterial occlusion or thrombosis.^5^, ^6^, ^7^

This recommendation, based on intuition and anecdotal data, has direct practical implications for many clinical scenarios. For instance, in patients presenting with spontaneous coronary artery dissections (SCAD) or stent edge dissections, and in those undergoing chronic total occlusion (CTO) intervention—where catheters are known to be often extraluminal in the vessel structure—it is recommended that repeated injections are avoided. Also, although the use of intravascular imaging is encouraged by guidelines when coronary dissections are suspected,^1^^,^^8^ optical coherence tomography (OCT) is considered a contraindication, by many, because contrast injections are necessary for image acquisition.^9^

In the present study, we sought to gain novel insights into the risk of contrast injection-induced coronary injury by analyzing invasive coronary hemodynamics during contrast injection at multiple points within the coronary artery and guiding catheter.

## Methods

### Study design and patient population

This is a prospective, single-arm, and multicenter study conducted at 4 centers in Brazil, Mexico, and the United Kingdom. Patients undergoing clinically indicated coronary pressure wire assessment during coronary angiography or percutaneous coronary intervention (PCI) were recruited. We included nonculprit coronary arteries without angiographic luminal stenosis or with non−flow-limiting stenoses, or culprit vessels after stenting physiologically significant lesions. Vessels with severe coronary artery disease were excluded. The study was given ethical approval locally at each center.

### Cardiac catheterization and pressure data recording

Cardiac catheterization and coronary angiography were undertaken via the radial or transfemoral routes at the operator’s discretion. A 6F guide catheter was used to cannulate the vessel of interest.

A pressure sensor tipped guidewire (Verrata Plus [Philips], Omniwire [Philips], PressureWire Aeris [Abbott], or PressureWire X [Abbott]) was used. After intracoronary administration of ≥200 μg of nitroglycerine, the pressure wire sensor was positioned at the guide catheter tip and the pressures equalized with the aortic pressure. The pressure sensor was then positioned at 5 predetermined locations for monitoring of contrast injections from distal to proximal: distal vessel, mid vessel, proximal vessel, at the catheter tip, and inside the catheter.

**Figure 1:**
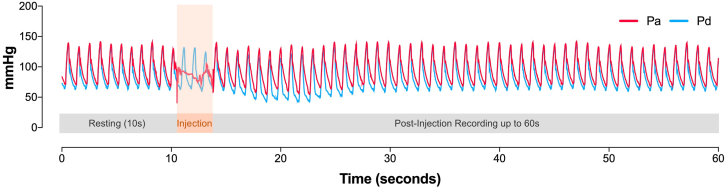
**Pressure recording protocol.** A continuous 60-second pressure recording was taken in each of the 5 predetermined locations (see text). The red tracing corresponds to the aortic pressure measured at the catheter tip (Pa). The blue tracing corresponds to the intracoronary pressure measured by the pressure wire sensor (Pd). Injections were taken after 10 seconds of resting recording and are defined by the loss of Pa waveform (orange shaded area). After injection, the recording was maintained for up to 60 seconds.

A continuous 60-second pressure recording was taken in each location, consisting of 10 seconds of resting recording, during contrast injection, contrast-induced hyperemia, and recovery of resting conditions (Figure 1). In 2 centers, contrast injections were randomized to be performed manually or with the use of an automatic injector pump. The automatic pump injections were recommended to be taken with a total contrast volume between 3 and 6 mL, flow rate between 3 and 4 mL/s, and injection pressure of 300 to 400 psi. These settings were not mandated and could be adjusted according to each operator’s discretion. In the remaining 2 centers, all contrast injections were performed manually.

### Data analysis

Angiographic images, coronary pressures, and aortic pressures were extracted from the instrument console and stored for offline analysis. Pressure data were analyzed offline using a custom software package designed with MATLAB (The Mathworks, Inc).

Peak systolic pressures remain relatively unchanged as they proceed along the coronary artery whereas end-diastolic pressures decrease steadily, thus gradually increasing the pulse pressure and decreasing the mean arterial pressure at more distal positions.^10^ For this reason, the peak systolic pressure was measured to assess the change in intracoronary pressure during contrast injections.

Temporary loss of the guiding catheter pressure reading (red pressure tracing in Figure 1) after 10 seconds of resting recording was used to demarcate the moment of contrast injection. The uninterrupted intracoronary pressure recording transmitted by the pressure wire sensor (blue pressure tracing in Figure 1) was used for calculating the peak pressure at resting baseline and during contrast injection.

Quantitative coronary angiography was performed at the Yale Angiographic Core Laboratory using validated software (QAngio XA version 7.3.102.0, Medis Medical Imaging). The distances from the pressure sensor relative to the guide catheter tip were determined at each measurement location. Reference vessel diameter in each vessel segment and the maximum percent stenosis diameter for the entire vessel length were measured at the end-diastolic phase.

### Study end points

The objective of this study is to evaluate whether contrast injections increase intracoronary pressures beyond resting levels and hence are likely to contribute to the propagation of coronary dissections when the entry point is away or close to the guiding catheter.

The primary end point was the change in intracoronary peak pressure between resting conditions (baseline) and during contrast injections in 5 predetermined locations. Absolute peak pressure during contrast injection was the secondary end point.

### Statistical analysis

Continuous variables are presented as mean ± SD. Peak coronary pressure at rest was compared to peak coronary pressure during contrast injection using a paired *t* test. To assess the difference between each vessel segment, absolute peak pressure during injection, and change in peak pressure from resting to injection at each segment were analyzed using a paired *t* test using the distal vessel as the reference. Results are presented as estimates with 95% CI. The difference in peak intracoronary pressure was plotted against the distance from the pressure sensor to the catheter. A line representing the smoothed moving median was plotted. Sensitivity analysis was performed to compare the difference in intracoronary pressure changes with manual and automatic injections (*t* test). A 2-sided *P* value <.05 was considered statistically significant. All data were analyzed using R version 4.2.3 (R Foundation for Statistical Computing). The function “movStats” was used from the “Hmisc” package for the moving-smoothed median.^11^

## Results

### Patient and vessel characteristics

A total of 52 patients (58 vessels, 271 contrast injections) were included. Data from 2 contrast injections were excluded due to the failure of the pressure sensor, leaving a total of 269 pressure recordings for analysis. Baseline patient clinical characteristics are summarized in Table 1. The mean age was 62.1 ± 9.4 years, and most (70.9%) patients presented with chronic coronary syndromes. The baseline vessel and procedural characteristics are summarized in Table 2. The left anterior descending artery was the most frequently studied vessel (56.9%) and the mean proximal reference vessel diameter measured 3.17 ± 0.46 mm. Out of the total 269 contrast injections, 164 (61.0%) were performed manually and 105 (39.0%) were performed with the assistance of an automatic injector pump. The mean contrast volume dispensed per injection was 4.23 ± 1.10 mL (manual injection 3.79 ± 0.97 mL, pump injection 4.84 ± 0.97 mL).

**Table 1:** Baseline patient demographics and clinical characteristics.

Characteristics	(N = 52 patients)
Age, y	62.1 ± 9.4
Female sex	23 (41.8)
Height, cm	166.6 ± 9.2
Weight, kg	79 ± 16.1
Hypertension	41 (74.5)
Hypercholesterolemia	41 (74.5)
Diabetes mellitus	16 (29.1)
Current smoker	5 (9.1)
Former smoker	15 (27.3)
Previous myocardial infarction	9 (16.4)
Previous percutaneous coronary intervention	7 (12.7)
Previous coronary artery bypass grafting	1 (1.8)
Indication for coronary angiography	
Silent ischemia	10 (19.2)
Stable angina	30 (57.7)
Unstable angina	7 (13.5)
Non-ST elevation myocardial infarction	5 (9.6)
Indication for pressure wire assessment^a^	
Intermediate coronary artery disease	40 (76.9)
Post percutaneous coronary intervention	13 (25.0)
Serial or diffuse coronary artery disease	5 (9.6)
Left main assessment	2 (3.8)
Guide percutaneous coronary intervention in multivessel disease	2 (3.8)

Values are mean ± SD or n (%).

a More than 1 indication is possible per patient.

**Table 2:** Baseline vessel and procedural characteristics.

Characteristics	(N = 58 vessels)
Target vessel	
Left anterior descending artery	33 (56.9%)
Left circumflex artery	9 (15.5%)
Right coronary artery	16 (27.6%)
Quantitative coronary analysis	
Most significant diameter stenosis, %	35.7 ± 12.1
Distal vessel	
Reference vessel diameter, mm	2.05 ± 0.47
Distance of pressure sensor to guiding catheter tip, mm	77.75 ± 14.68
Mid vessel	
Reference vessel diameter, mm	2.70 ± 0.47
Distance of pressure sensor to guiding catheter tip, mm	45.33 ± 12.99
Proximal vessel	
Reference vessel diameter, mm	3.17 ± 0.46
Distance of pressure sensor to guiding catheter tip, mm	17.62 ± 7.27
Guiding catheter tip	
Reference vessel diameter, mm	3.91 ± 0.68
Distance of pressure sensor to guiding catheter tip, mm	–0.01 ± 2.05^a^
Inside the catheter	
Distance of pressure sensor to guiding catheter tip, mm	–23.85 ± 7.83^a^
Contrast injection mode	N = 269 recordings
Manual	164 (61.0%)
Contrast volume, mL	3.79 ± 0.97
Injector pump	105 (39.0%)
Contrast volume, mL	4.84 ± 0.97
Pressure, psi	394 ± 140.7
Flow rate, mL/s	4.19 ± 0.68

Values are mean ± SD or n (%).

a Negative values indicate distances from the pressure sensor inside the catheter to the catheter tip.

### Changes in peak pressure during injections within the vessel and inside the guiding catheter

**Figure 2:**
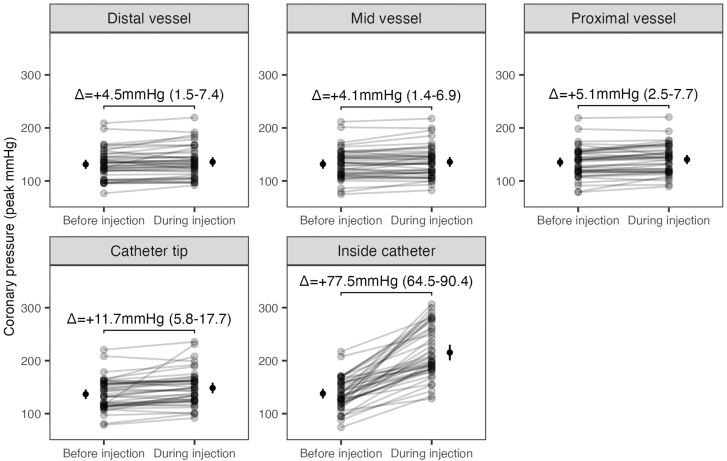
**Peak pressure at resting and during injection at each coronary segment.** Changes in peak pressure between resting and during injection for each analyzed recording in each vessel location. Numbers are the mean difference between resting and during injection with 95% CI.

**Figure 3:**
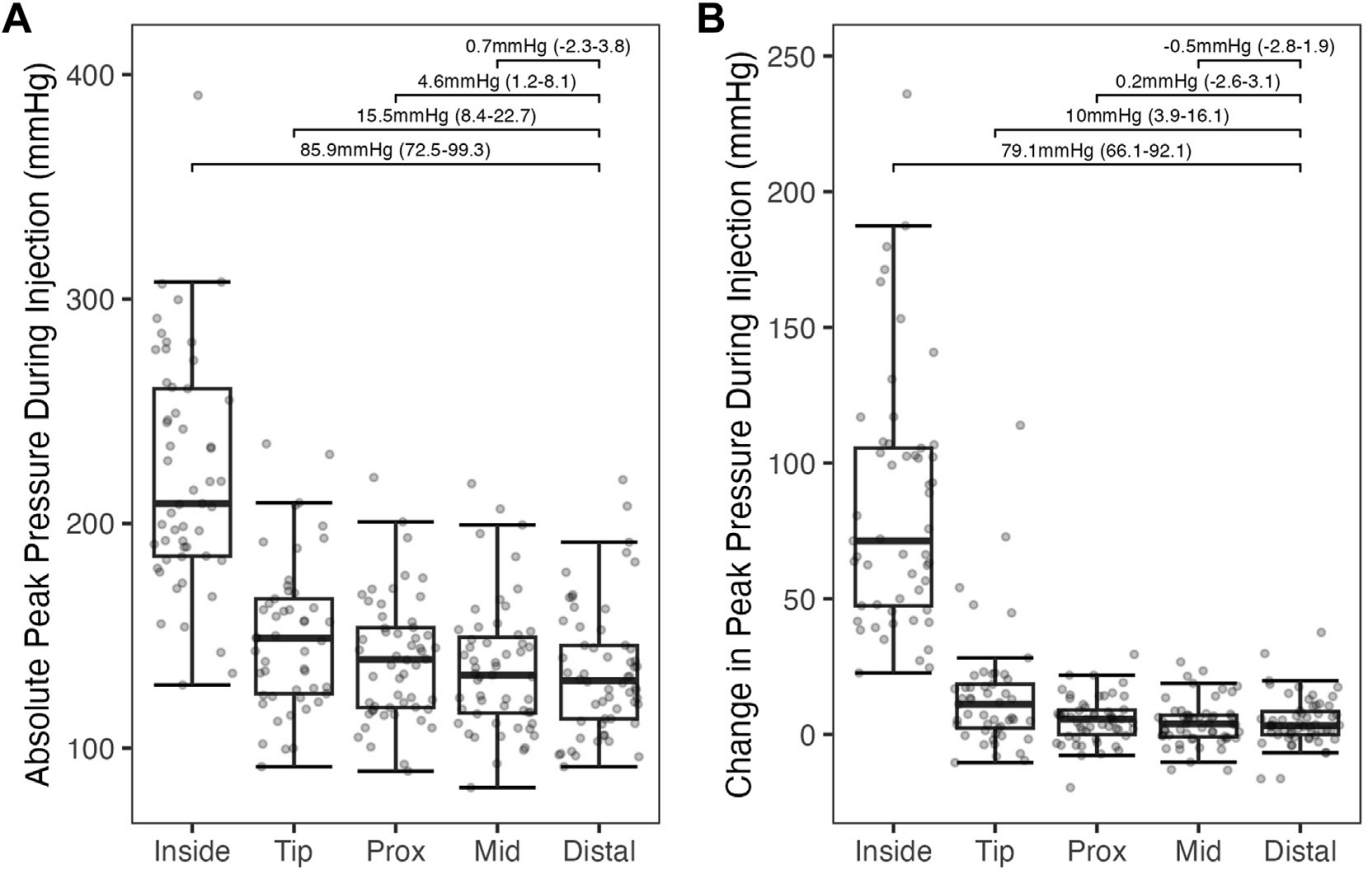
**Peak absolute pressure and pressure differences during contrast injection at each coronary segment.** (**A**) The absolute peak pressure during injection at each coronary location. Considering the distal vessel location as a reference, significant differences in peak pressure were seen between the proximal vessel, catheter tip, and inside the catheter. (**B**) Differences in peak pressure from resting to during injection at each coronary location. Considering the distal vessel as a reference, significant differences in peak pressure were seen in the catheter tip and inside the catheter.

Compared to resting, contrast injection led to a small increase in peak coronary pressures in distal, mid, and proximal segments of coronary vessels (distal vessel mean peak pressure difference +4.5 mm Hg; 95% CI, 1.5-7.4; mid vessel mean peak pressure difference, +4.1 mm Hg; 95% CI, 1.4-6.9; and proximal vessel mean peak pressure difference, +5.1 mm Hg; 95% CI, 2.5-7.7). Change in peak pressure during injection was higher at the catheter tip (mean peak pressure difference, +11.7 mm Hg; 95% CI, 5.8-17.7) and significantly greater inside the catheter (mean peak pressure difference, +77.5 mm Hg; 95% CI, 64.5-90.4) (Table 3 and Figure 2). When using the distal vessel as the reference, changes in peak pressure caused by injection were significantly greater at the catheter tip (10 mm Hg, *P* < .01) and inside the catheter (79.1 mm Hg, *P* < .01) (Figure 3).

**Table 3:** Change in intracoronary peak pressure from resting to during injection.

Vessel location	Resting peak pressure (mm Hg)	Peak pressure during injection (mm Hg)	Difference (95% CI)	*P* value
Inside catheter	137.9 ± 30.0	215.3 ± 47.8	+77.5 (64.5-90.4)	<.01
Catheter tip	136.7 ± 29.5	148.5 ± 33.0	+11.7 (5.8-17.7)	<.01
Proximal vessel	135.2 ± 28.4	140.4 ± 27.7	+5.1 (2.5-7.7)	<.01
Mid vessel	131.7 ± 28.5	135.8 ± 29.6	+4.1 (1.4-6.9)	<.01
Distal vessel	131.3 ± 27.7	135.8 ± 29.2	+4.5 (1.5-7.4)	<.01

Values are mean ± SD or n (%). Differences are peak pressure during injection minus peak pressure at rest.

### Injection-induced pressure changes and distance from the guiding catheter

**Figure 4:**
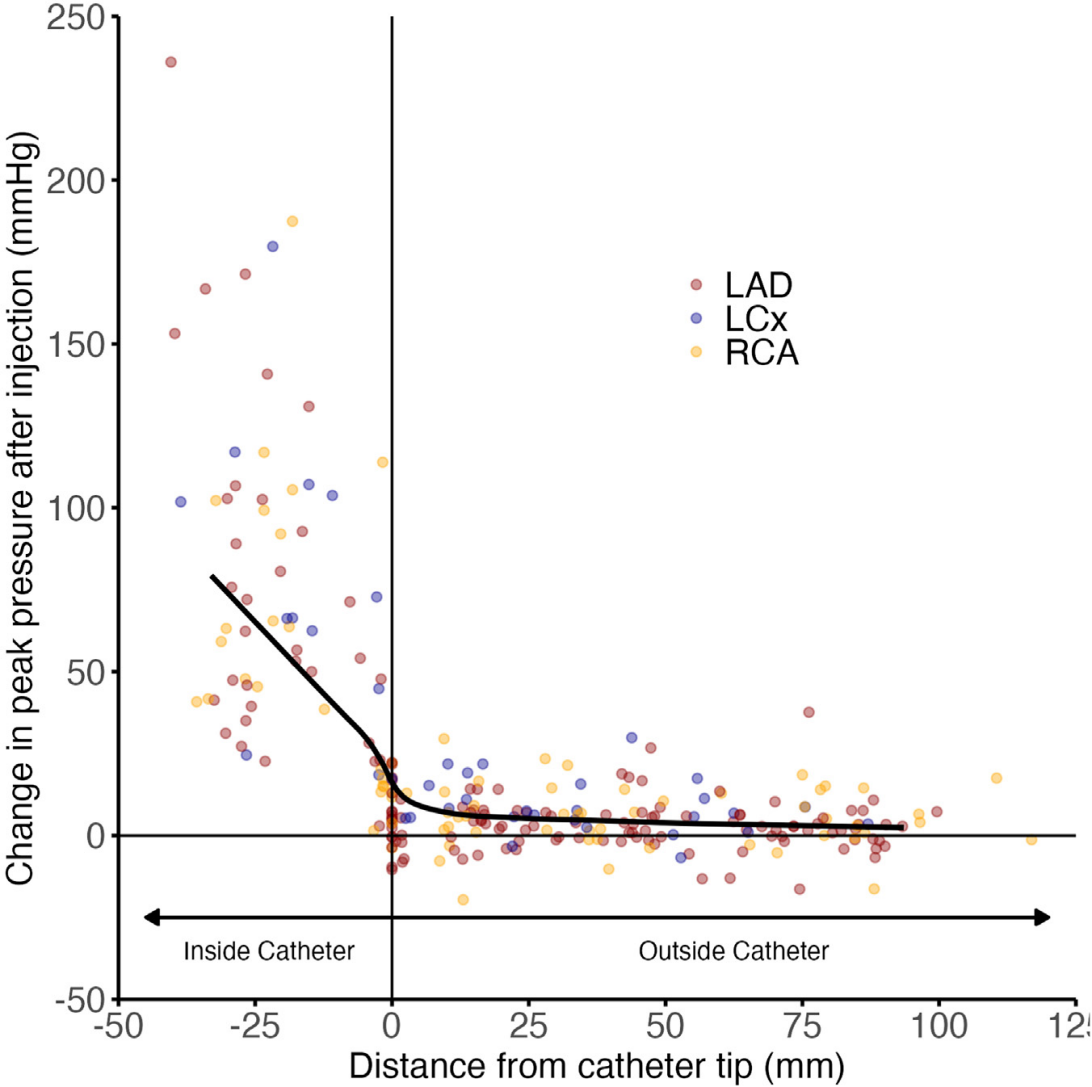
**Peak pressure difference according to distance from the catheter tip.** Scatterplot displaying the difference in peak pressure from resting to during injection as a function of the distance from the catheter tip. The data are fitted with a line demonstrating a smoothed moving median. LAD, left anterior descending artery; LCx, left circumflex artery; RCA, right coronary artery.

**Figure 5:**
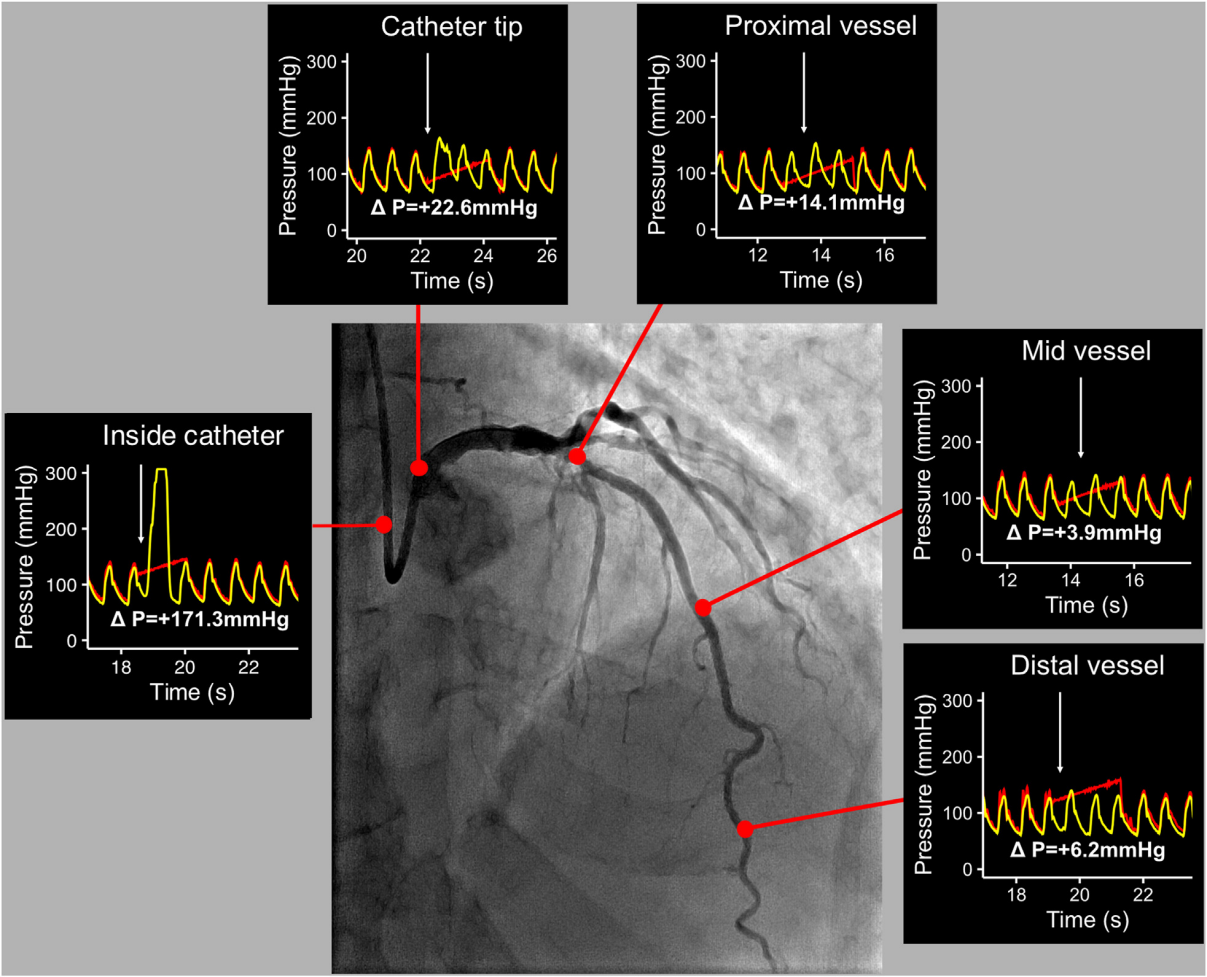
**Case example demonstrating the changes in peak pressure during injection at each coronary location.** Red traces are the aortic pressure (Pa) transmitted by the guiding catheter, and the yellow traces are the intracoronary pressure (Pd) transmitted by the pressure wire. The white arrow shows the point of injection. Note the significant increase of Pd during injection with loss of its normal waveform morphology when inside the catheter and the catheter tip. Note the negligible differences in the Pd during injection and at resting when the pressure sensor is in the other coronary segments.

Irrespective of vessel segment categorization (distal, mid, or proximal), the closer the measurements were to the guiding catheter tip, the higher were the pressure changes caused by contrast injections (Figure 4). Larger magnitudes of peak pressure changes caused by contrast injection were only consistently observed inside the catheter and, to a lesser extent, at the level of the catheter tip. Importantly, although beyond the catheter tip, the typical coronary waveform is not altered by contrast injection, it is completely lost inside the catheter with a sudden upright surge caused by injection forces (Figure 5).

Distal to the catheter tip, changes in pressure caused by injection are of much smaller magnitude than the changes observed within each beat (delta between diastolic nadir and systolic peak pressures). The average dp/dt before contrast injection was 234.0 ± 80.0 mm Hg/s. This increased to 286.6 mm Hg ± 110.0 mm Hg/s during injection at the catheter tip and 303.6 ± 96.4 mm Hg/s inside the catheter. No change in the pressure waveform was seen at the proximal, mid, and distal vessel positions during injection.

### Effects of type of injection and target vessel

**Figure 6:**
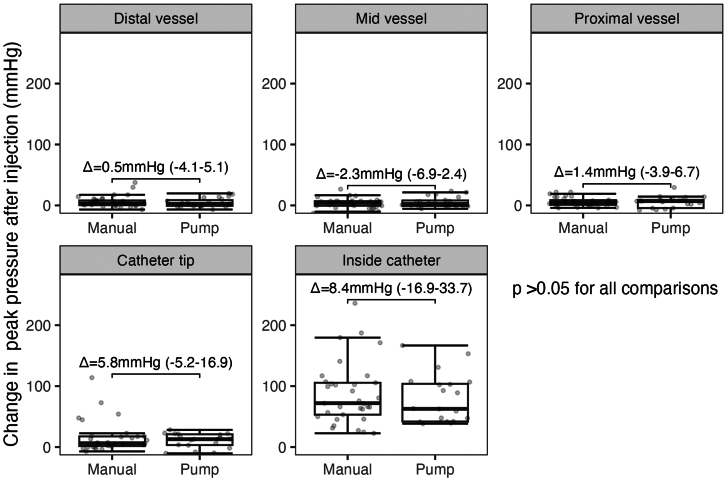
**Peak pressure at resting and during injection at each coronary segment according to the method of contrast injection.** Changes in peak pressure between resting and during injection in each vessel location according to the type of injection (manual vs automatic pump injectors). Numbers are the mean difference between resting and during injection with 95% CI.

No significant differences were found in the changes observed in peak pressures between injections performed manually or using automatic injector pumps across all vessel locations (Figure 6). The relationship between injection and change in peak pressure at the 5 predetermined locations was not influenced by the vessels assessed (Figure 4).

## Discussion

The current study presents the most contemporary and comprehensive assessment of the effects of contrast injection on coronary hemodynamics, offering unique insights into the risks of injection-induced vessel injury. We found that although manual and pump contrast injections consistently cause a significant change in pressures inside the guiding catheter, they only lead to negligible changes in pressures inside the coronary artery, beyond the guiding catheter tip (Central Illustration and Figure 3, Figure 4, Figure 5). Although statistically significant when compared to resting, the pressure changes observed during injections were very small in the proximal, mid, and distal segments of all 3 vessels (Table 3, Figure 2). Such values fall well within the known beat-to-beat variability of normal coronary pressures.^12^ Therefore, our data demonstrate that although some hydraulic force is needed to overcome the resistance imposed by the catheter, no residual force is significantly transmitted beyond the catheter tip, into the coronary artery itself.

**Central Illustration:**
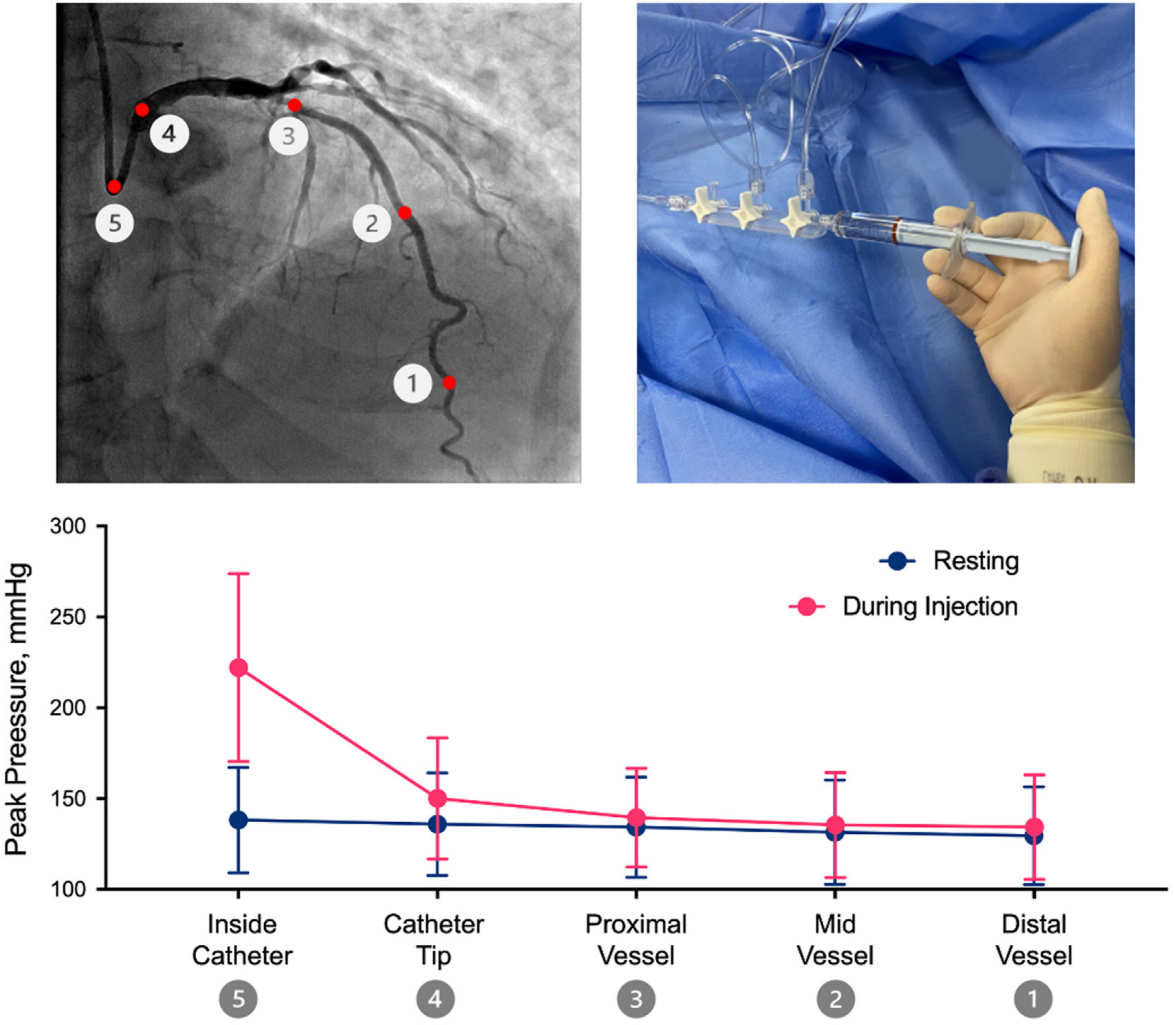
**Effects of contrast injections on intracoronary hemodynamics.** Although contrast injections significantly increase peak pressures inside the guiding catheter, they only lead to negligible changes in pressures inside the coronary artery, beyond the guiding catheter tip.

### Clinical implications: Vessel injury during routine coronary angiography

Our results have practical implications as to how we should perceive the risk of injection-induced vessel injury. First, our data suggest that intracoronary injections can only cause or propagate dissections close to the catheter, or, even more so, if the catheter tip is inside the vessel (or false lumen) with signs of pressure damping. Repeated and forceful injections should therefore be avoided when the area at risk is close to the catheter or when there are changes in aortic pressure waveform (pressure damping or ventricularization) suggesting catheter overengagement and/or ostial disease. Dissections that occur beyond the proximal vessel segment, such as distal stent edge dissections or SCAD should not be at risk of worsening with repeated injections, and consequently, contrast injection can be considered if clinically needed.^13^ The risk of propagating a dissection occurring beyond the proximal vessel segment during a SCAD assessment is more likely related to the advancement of wires and imaging catheters along the false lumen rather than to the injection of contrast media itself.^14^ Figure 5 shows a typical example of the pressure changes caused by injections observed within a coronary artery and inside the catheter.

It is important to emphasize our analysis does not imply that pressure changes generated by contrast injections are the only mechanism responsible for causing or propagating vessel injuries. Causation of spontaneous or iatrogenic coronary dissections is multifactorial and many other elements play important roles in its pathophysiology, such as vessel wall fragility, shear stress, and vessel tortuosity. Our study simply intends to isolate the potential contributions of contrast injections on top of all other elements and risks, by quantifying the transmission of hydraulic forces to multiple vessel locations.

### Choice of intravascular imaging modality when dissection is suspected

Optical coherence tomography imaging acquisition requires injection of greater contrast volumes than those used during standard angiography—usually 10 to 14 mL delivered over 3.0 to 4.0 seconds, either manually or with an injector pump.^15^^,^^16^ Therefore, our study findings should be extrapolated with caution to the scenario of OCT usage.

First, the total contrast volume will dictate how long the injection will last and is adjusted by the flow rate (mL/s). Second, coronary injections are harder during OCT acquisitions because the resistance is higher inside the guiding catheter, as the presence of the OCT catheter reduces its lumen. The total contrast volume will only influence how long the injection pressure is applied. The average pressure (394 psi) and flow rate (4.19 mL/s) used for the injector pump-controlled angiographies in our study are within the ranges of pressure and flow rates used for OCT acquisitions.^15^^,^^16^ Our study demonstrated that such pressures are not transmitted beyond the catheter tip and do not propagate significantly downstream the coronary vessel, regardless of whether manual or pump injection is used (Figure 6). Similarly, Shimamura et al^17^ found that intracoronary pressure increases were small during contrast injection for coronary angiography and OCT image acquisitions, in a single midvessel location.

Therefore, our results suggest that OCT is a safe imaging modality to assess patients with suspected coronary dissections or injuries, provided that they are not near the catheter tip. For catheter-induced, ostial, or very proximal dissections, or when there are signs of pressure waveform damping, it would be sensible to use intravascular ultrasound over OCT as the imaging modality of choice.

### Implications for CTO PCI

In CTO PCI, injection of contrast media into a closed extraluminal compartment is believed to increase the risk of extending a subintimal/intramural hematoma by exposing the false lumen to above-average pressures. Although our data were not acquired during CTO procedures, our findings of extreme peak pressure changes inside the catheter (up to 390.75 mm Hg) support the recommendation of avoiding injections via antegrade catheters in such procedures.^18^ It is plausible that injection pressures could be transmitted to an enclosed extraluminal compartment, compressing the vessel lumen.

### Potential mechanisms of injection-induced ventricular arrhythmias

Direct contrast injection into a small coronary vessel (such as the conus branch or a venous graft) is known to cause ventricular arrhythmias and cardiac arrest.^6^ Such arrhythmias usually initiate precisely at the time of peak injection. Our results suggest that extreme transmission of pressure into the coronary circulation could be a potential mechanism of arrhythmia initiation. Therefore, in situations where the catheter completely occludes a coronary artery or branch, as indicated by significant changes in pressure waveform morphology (eg, pressure damping or ventricularization), injections should be avoided, or at least performed with minimal injection forces if the need for vessel visualization outweighs the risks.

### Limitations

This study aimed to indirectly determine potential risks of coronary vessel injury during contrast injection by quantifying the changes in intracoronary pressure caused by manual and pump injections. Our population did not include patients with documented or suspected dissections and consisted of patients with anatomically mild to moderate coronary artery disease. We believe it would be ethically controversial to perform such a study in a population of patients with coronary dissection before understanding the changes in intracoronary pressure during contrast injection in nondissected vessels. Equally, data were not acquired during intravascular imaging acquisition with OCT, although it is reasonable to assume that the same principles should apply with or without intracoronary instrumentation.

We only analyzed data with normal aortic pressure waveforms. Therefore, our data should not be extrapolated to situations where proximal coronary pressure is damped or ventricularized. In such cases, the high pressures generated inside the catheter during injection could likely be transmitted further downstream.

To ensure unobstructed transmission of hydraulic forces generated during contrast injection, we only analyzed nonobstructed vessels or vessels with mild and nonfunctionally significant obstructions. However, it is not expected that vessels with obstructive atherosclerotic disease would behave differently. Although the presence of obstructive epicardial coronary disease would attenuate the distal transmission of peak pressures from the catheter (not increase), clinicians need to be vigilant for the presence of ostial disease and pressure damping, which could, conversely, increase transmission of pressures distally.

## Conclusions

The hydraulic forces needed for contrast media injection during percutaneous procedures do not lead to a clinically significant increase in coronary pressures beyond the segments very close to the coronary catheter. Although care should be taken during injections in cases of very proximal dissections or with catheter-induced pressure damping, it is unlikely that routine contrast injections would cause or worsen coronary injuries beyond the region close to the catheter.
